# Nanoscale investigation of enhanced electron field emission for silver ion implanted/post-annealed ultrananocrystalline diamond films

**DOI:** 10.1038/s41598-017-16395-1

**Published:** 2017-11-24

**Authors:** Kalpataru Panda, Jeong Jin Hyeok, Jeong Young Park, Kamatchi Jothiramalingam Sankaran, Sundaravel Balakrishnan, I.-Nan Lin

**Affiliations:** 1 Center for Nanomaterials and Chemical Reactions, Institute for Basic Science (IBS), Daejeon, 34141 Korea; 2 Graduate School of EEWS, Korea Advanced Institute of Science and Technology (KAIST), Daejeon, 34141 Korea; 3 Institute for Materials Research (IMO), Hasselt University, 3590 Diepenbeek, Belgium; 4 IMOMEC, IMEC vzw, Diepenbeek, Belgium; 5 Materials Physics Division, Indira Gandhi Centre for Atomic Research, Kalpakkam, 603 102 India; 6 Department of Physics, Tamkang University, Tamsui, 251 Taiwan, ROC

## Abstract

Silver (Ag) ions are implanted in ultrananocrystalline diamond (UNCD) films to enhance the electron field emission (EFE) properties, resulting in low turn-on field of 8.5 V/μm with high EFE current density of 6.2 mA/cm^2^ (at an applied field of 20.5 V/μm). Detailed nanoscale investigation by atomic force microscopy based peak force-controlled tunneling atomic force microscopy (PF-TUNA) and ultra-high vacuum scanning tunneling microscopy (STM) based current imaging tunneling spectroscopy (CITS) reveal that the UNCD grain boundaries are the preferred electron emission sites. The two scanning probe microscopic results supplement each other well. However, the PF-TUNA measurement is found to be better for explaining the local electron emission behavior than the STM-based CITS technique. The formation of Ag nanoparticles induced abundant sp^2^ nanographitic phases along the grain boundaries facilitate the easy transport of electrons and is believed to be a prime factor in enhancing the conductivity/EFE properties of UNCD films. The nanoscale understanding on the origin of electron emission sites in Ag-ion implanted/annealed UNCD films using the scanning probe microscopic techniques will certainly help in developing high-brightness electron sources for flat-panel displays applications.

## Introduction

Nowadays electron sources based on the field emission (FE) concept, namely “cold-cathodes”, are substituting the conventional thermionic electron sources. FE based cold cathode devices are characterized by their reduced weight, instantaneous switching on properties, capability to operate at high frequency/high current densities and operation without any outside heating element for the emission process contrary to their thermionic based electron sources^1–3^. The performance of such FE based cold cathode devices are further improved by using nanostructured materials as field emitters. Over the last few years, the design, realization, and applications of such nanostructured based cold cathode devices have been the object of tremendous interest by the research communities. Among the most extensively investigated nanostructured materials for cold cathode applications, carbon nanotubes^4–7^, graphene^8‒11^and graphdiyne^12,13^ have a prominent place, because of their superior FE properties. However, these nanocarbon based electron field emission (EFE) materials face the challenge of insufficient lifetime stability^4–13^. Recently, ultra-nanocrystalline diamond (UNCD) materials have gained much attention because of their high lifetime stability^1–3,6^ with superior EFE properties^6,14‒16^. The use of UNCD for the fabrication of cold cathode emitter/electron emitting devices requires the film to be conductive. Previous studies showed that the variation of chemical bonding structure, doping by foreign dopants (*e*.*g*. nitrogen, boron, phosphorus), metallic film coating/annealing, interlayer modifications, surface hydrogenation, and synthesizing hybrid-nanostructured diamond play a significant role in improving the conductivity/EFE properties for diamond and related materials^14‒22^. Metallic species (*e*.*g*. Li, Cs, Ca, Sr and Ba) have also been doped into the diamond matrix to lower the threshold value for electron emission, but the EFE properties are still not satisfactory^23^.

Facilitating the formation of nanographitic grain boundary phases by addition of N_2_ into the plasma during UNCD film growth can improve the electrical conductivity and enhance the EFE properties^24–26^. However, a high growth temperature of about 800 °C is required to activate the N_2_ in the UNCD films^25,26^^.^ The doping of foreign ions using the well-known ion implantation technique can modify the properties of UNCD materials through controlled incorporation of a variety of dopants^27–29^. Ion implantation can break the C–C, sp^3^, and hydrocarbon bonds to form a sp^2^-rich graphitic phase, introduce defects, and can be used to tailor the sp^2^/sp^3^ ratio for diamond and related materials^28,29^. Conversion of the amorphous carbon (a-C) phases present in the grain boundaries to a graphitic phase seem to be a plausible explanation for the improved electrical conductivity and EFE properties of UNCD films. However, few groups have attempted to find out the exact electron emission sites to directly correlate the role of the diamond/non-diamond phases on the EFE properties at local scale.

Scanning tunneling microscopy (STM) is being utilized to investigate the local EFE behavior and surface electronic properties of doped diamond-like carbon and diamond films^30–33^. Krauss and co-workers used STM to show that the emission sites from UNCD-coated Si tips are related to the grain boundaries in the UNCD surface topography, and are not related to surface asperities or grains^34^. By using STM, Karabutov *etal*. explained that the sharp morphological protrusions (grains) of microcrystalline diamond films are not the real emission sites, but rather the grain boundaries^35^. Static and dynamic STM experiments were carried out to show that the grain boundaries of UNCD are the prominent electron emission sites^36–38^. However, the STM results did not show good enough contrast to clearly illustrate the distribution of the local electron emission sites. Hence, to reveal the mechanism behind the enhanced conductivity/EFE properties of the UNCD films, an experimental investigation that can directly correlate the nanometer-scale electron emission sites with their microstructure is required.

Over the past few years, advances in both STM and atomic force microscopy (AFM) have developed the conducting_-_AFM/STM combination into a highly sensitive technique called tunneling atomic force microscopy (TUNA)^39–42^. In contrast to standard STM-based current imaging tunneling spectroscopy (CITS) measurements that require the sample surfaces to be smooth at nanometer scale, TUNA can investigate surfaces with a root mean squared (RMS) roughness of several micrometers, which allows a wide picture of the overall morphology to be scanned. Moreover, in contrast to constant-current mode STM, physical tracking of the sample surface in TUNA means that the height data collected from the deflection of the cantilever avoids possible artifacts introduced by variations in the conductivity of the sample surface. Another major advantage of TUNA is that it has a very high current sensitivity with a current measurement range up to 120 pA and a noise level of 50 fA. This allows for electrical characterization of undoped/unterminated diamond samples at high lateral resolution in contrast to standard STM measurements that require the sample surface to be sufficiently conductive^39^. It should be noted that TUNA is not the same as conducting AFM where the tip is always in contact with the sample surface that can change the tip condition and may not always show the true electronic properties of the sample surface.

In this context, by using Ag-ion implanted/post-annealed UNCD films as model materials, the local electron emission sites from these films was directly explored by using the AFM-based PF-TUNA technique. In the meantime, the microstructure of the samples was investigated using transmission electron microscopy (TEM). The mechanism behind the enhanced conductivity/EFE properties was investigated by correlating the PF-TUNA and TEM observations. Moreover, the spectroscopic results from AFM-based PF-TUNA and STM-based CITS were compared to illustrate the overwhelming advantage of the PF-TUNA measurements over STM-CITS in investigating the local electron emission behavior.

## Results

### Material and electrical characteristics

**Figure 1:**
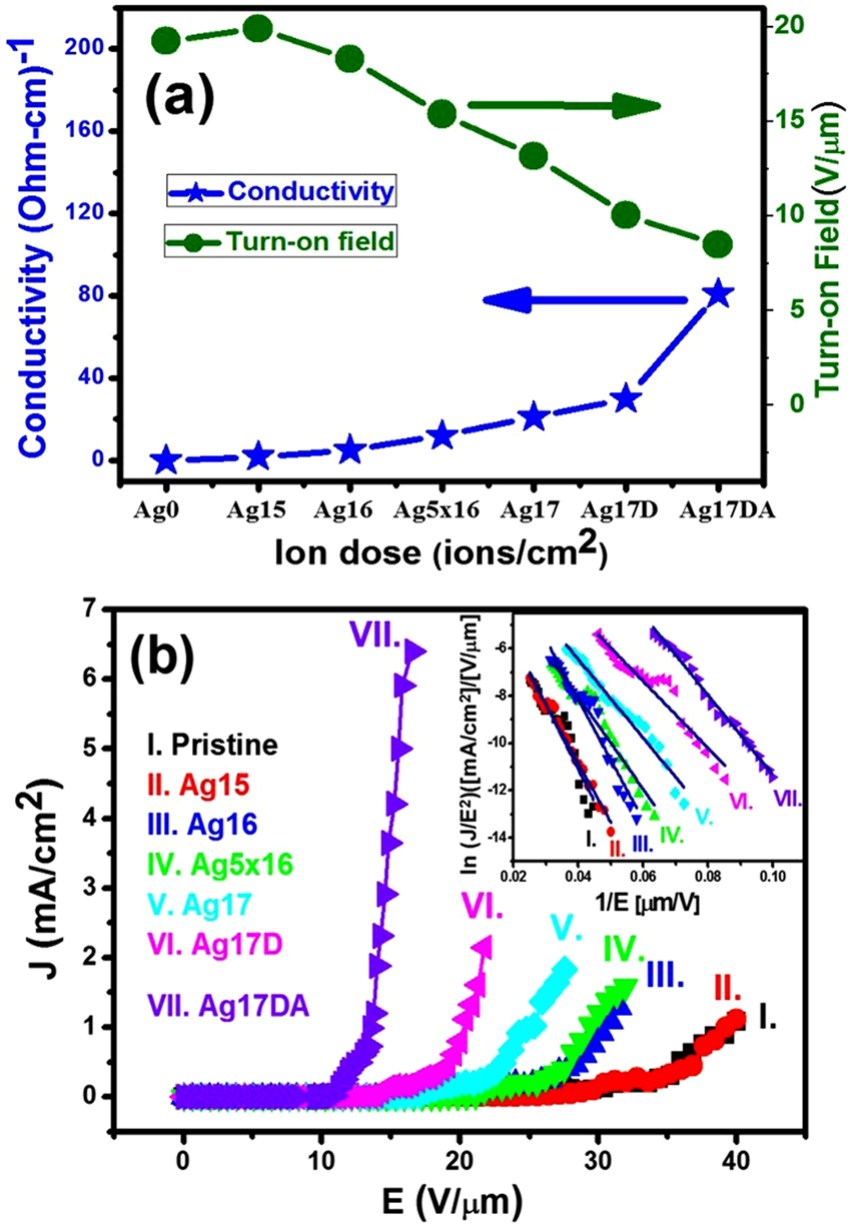
**(a**) Variation in the electrical conductivity (solid stars) and turn-on field (closed circles) as a function of the dosage of Ag ions implanted in the UNCD films. (**b**) The electron field emission properties of various dosages of Ag-ion implanted and post-annealed UNCD films. The inset of (**b**) shows the corresponding Fowler Nordheim (F‒N) plots.

The marked changes on the surface microstructure of pristine UNCD (Ag0) films via the Ag-ion implantation (Ag17D) and Ag-ion implantation/post-annealing (Ag17DA) processes are shown in the FESEM analysis in Fig. S1 of the supplementary information. The electrical conductivity of Ag-ion implanted/post-annealed UNCD films determined from the Hall measurements in the van der Pauw configuration is plotted in Fig. 1a (closed star symbols) against the ion dosage. It shows that the Ag0 and Ag15 films are too resistive for the Hall measurements, whereas the electrical conductivity of the Ag-ion implanted UNCD films increases monotonically with increasing the ion dosages, from 5 (Ohm‒cm)^−1^ for Ag16 to about 30.0 (Ohm‒cm)^−1^ for the Ag17D films. The conductivity further increases to about 78.0 (Ohm‒cm)^−1^ with a sheet carrier concentration of n = 1.2 × 10^18^ cm^−2^ and mobility of µ = 9.0 × 10^3^ cm^2^ V^−1^ s^−1^ for the Ag17DA films.

Moreover, the higher the conductivity the films are, the better the EFE properties. Figure 1b shows that the pristine and Ag15 UNCD films need a large field to turn on the EFE process, (E_0_)_Ag0_  = 19.5 V µm^−1^ and (E_0_)_Ag15_  = 19.91 V µm^−1^, with a small EFE current density (i.e. J < 1.0 mA cm^−2^) at an applied field of 40.0 V µm^−1^ (curves I and II, respectively). The turn-on field required to initiate the EFE process decreases monotonically with the Ag-ion implantation process dosage, viz. from (E_0_)_Ag16_  = 18.29 V µm^−1^ with J = 1.28 mA cm^−2^ (at 35.5 V µm^−1^) for Ag16 films to (E_0_)_Ag17D_  = 10.05 V µm^−1^ with J = 2.15 mA cm^−2^ (at 21.7 V µm^−1^) for Ag17 films and then to (E_0_)_Ag17DA_  = 8.5 V µm^−1^ with J = 6.2 mA cm^−2^ (at 20.5 V µm^−1^) for Ag17DA films. These electrical conductivity and EFE characteristics are summarized in Table 1, which reveal that only the Ag17D and Ag17DA films exhibit sufficiently good electrical conductivity and EFE properties. The characteristics of these films were further analyzed to understand the contributing factor that improved the conductivity/EFE properties.

**Table 1:** Electrical and electron field emission properties of Ag-ion implanted/post-annealed UNCD films.

Samples	Sheet carrier concentration (cm^−2^)	Mobility (cm^2^ V^−1^ s^−1^)	Conductivity (Ω cm)^−1^	Turn-on field (V µm^−1^)	Current density (mA cm^−2^)
Ag15	—	—	—	19.91	0.004 @ 23.2 V/µm
Ag16	7.3 × 10^14^	0.3 × 10^1^	5	18.29	1.28 @ 35.5 V/µm
Ag5E16	2.9 × 10^15^	1.1 × 10^1^	12	15.38	1.57 @ 31.8 V/µm
Ag17	2.7 × 10^16^	8.3 × 10^1^	21	13.17	1.8 @ 27.6 V/µm
Ag17D	3.2 × 10^17^	7.9 × 10^2^	30	10.05	2.15 @ 21.7 V/µm
Ag17DA	1.2 × 10^18^	9 × 10^3^	78	8.5	6.2 @ 20.5 V/µm

**Figure 2:**
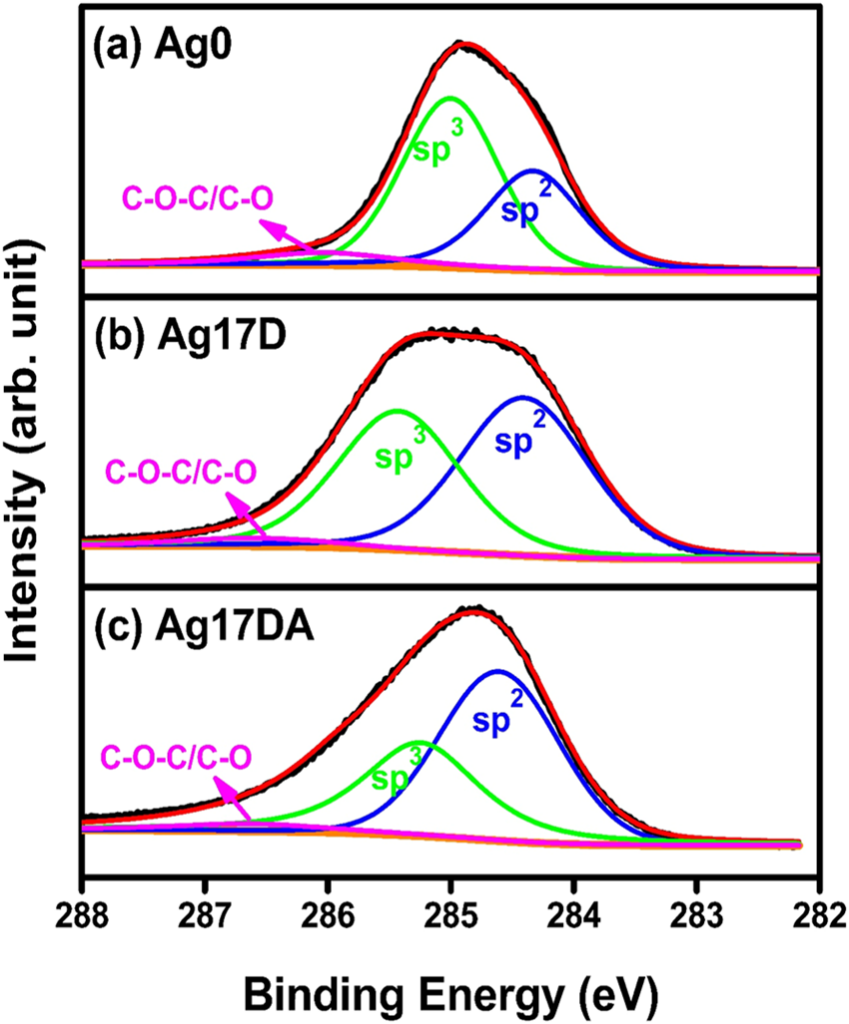
C1s XPS spectra of the (**a**) Ag0, (**b**) Ag17D, and (**c**) Ag17DA films.

XPS measurements were carried out to understand the effect of Ag-ion implantation and the subsequent post-annealing processes on the surface chemical bonding characteristics of the UNCD films. C 1 s photoemission spectra of the Ag0, Ag17D, and Ag17DA films are shown in Fig. 2a,b and c, respectively, where the background was subtracted using Shirley’s method^43^. The data were fitted with Lorentzian peaks with binding energies at 284.2, 285.3, and 286.4 eV corresponding to the sp^2^ (C=C), sp^3^ (C–C), and C–O–C/C–O bonds; their relative intensities are tabulated in Table 2. In the Ag0 films (Fig. 2a), sp^3^ bonding is dominant with a peak intensity of 60.3%, while the sp^2^ intensity is 36.5%, and a small amount of C–O–C/C–O has an intensity of 3.2%. Figure 2b shows that, after Ag-ion implantation of 1.74 × 10^17^ ions cm^−2^ (i.e., A17D films), the sp^2^ peak intensity increased to 53.4% while the sp^3^ peak intensity decreased to 44.2%. Figure 2c indicates that post-implantation annealing of these films further increased the sp^2^ phase content to 61.8% while the sp^3^ intensity decreased to 36.4% for Ag17DA films. The Ag-ion implantation/post-annealing process was observed to increase the sp^2^ phase content for the UNCD films.

**Table 2:** Relative intensities of various components of the C1s XPS spectra from Ag0, Ag17D and Ag17DA.

Peak position	Chemical bonding	Samples Peak intensity (%)		
(eV)		Ag0	Ag17D	Ag17DA
284.2	sp^2^ (C = C)	31.5	38.5	59.1
285.2	sp^3^ (C-C)	63.6	55.5	34.8
286.2	C-O/C-O-C	4.9	6.0	6.1

**Figure 3:**
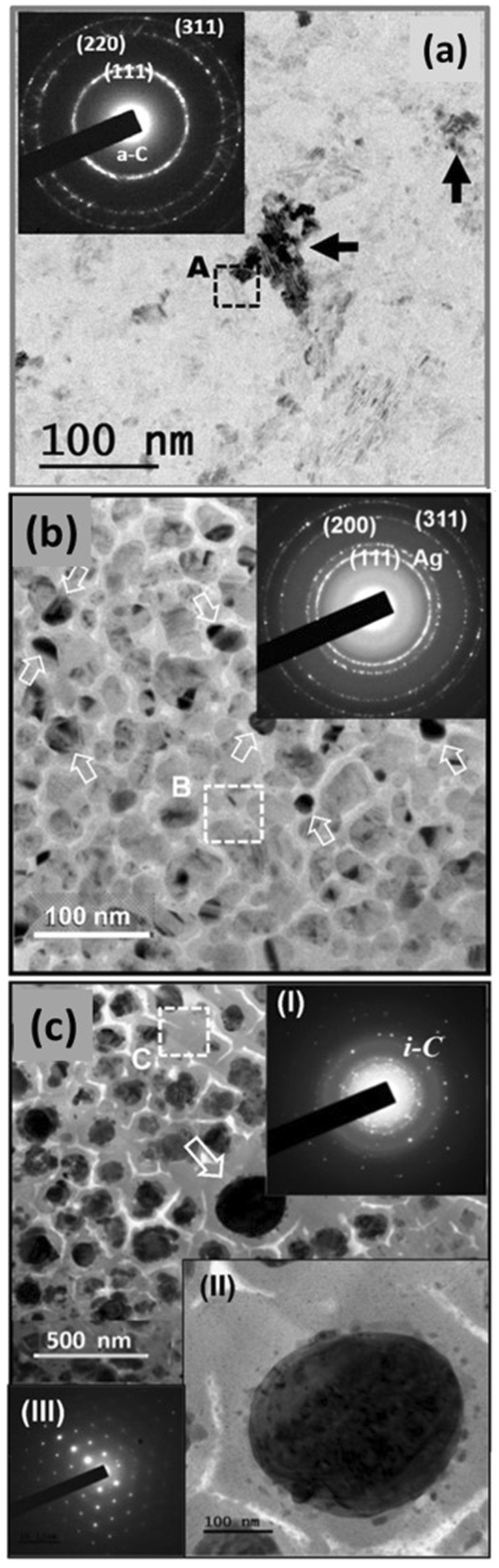
Bright field TEM image of (**a**) Ag0, (**b**) Ag17D and (**c**) Ag17DA films with their corresponding SAED patterns shown in the respective insets. Insets II and III in (**c**) show the bright-field TEM microstructure and corresponding SAED of the large Ag nanoparticle (marked by the arrow).

These results clearly illustrate the beneficial effect of Ag-ion implantation/annealing processes on enhancing the conductivity and EFE properties of UNCD films; the change in microstructures due to these processes is expected to be a key factor. To explore this phenomenon, more-detailed TEM examinations were carried out. Figure 3a shows the bright field TEM (BF-TEM) microstructure of pristine UNCD (Ag0) films with their corresponding selected area electron diffraction (SAED) pattern shown in the inset. The pristine UNCD films (Ag0) possess nano-sized diamond grains with large diamond aggregates occasionally observed (indicated by arrows). The ultra-small diamond grains are too small to be resolved in Fig. 3a, but the existence of these diamond grains is inferred by the corresponding SAED pattern (inset, Fig. 3a), which contains sharp diffraction rings corresponding to the (111), (220), and (311) diamond lattices. No diffraction ring for a material other than diamond was observed. Moreover, the central diffused ring, corresponding to sp^2^-bonded carbon (graphitic or a-C phase) is not very prominent in the SAED pattern (inset, Fig. 3a). This implies that sp^2^-bonded carbon in these films does not make up a large proportion of the content. The high-resolution TEM (HRTEM) micrograph, which is not shown here, indicates that the a-C phase is located along the grain boundaries. Moreover, the large diamond aggregates  (Fig. 3a) seem to be a soft agglomeration of small diamond grains, as it can be easily disintegrated by the electron irradiation during the TEM examination.

**Figure 4:**
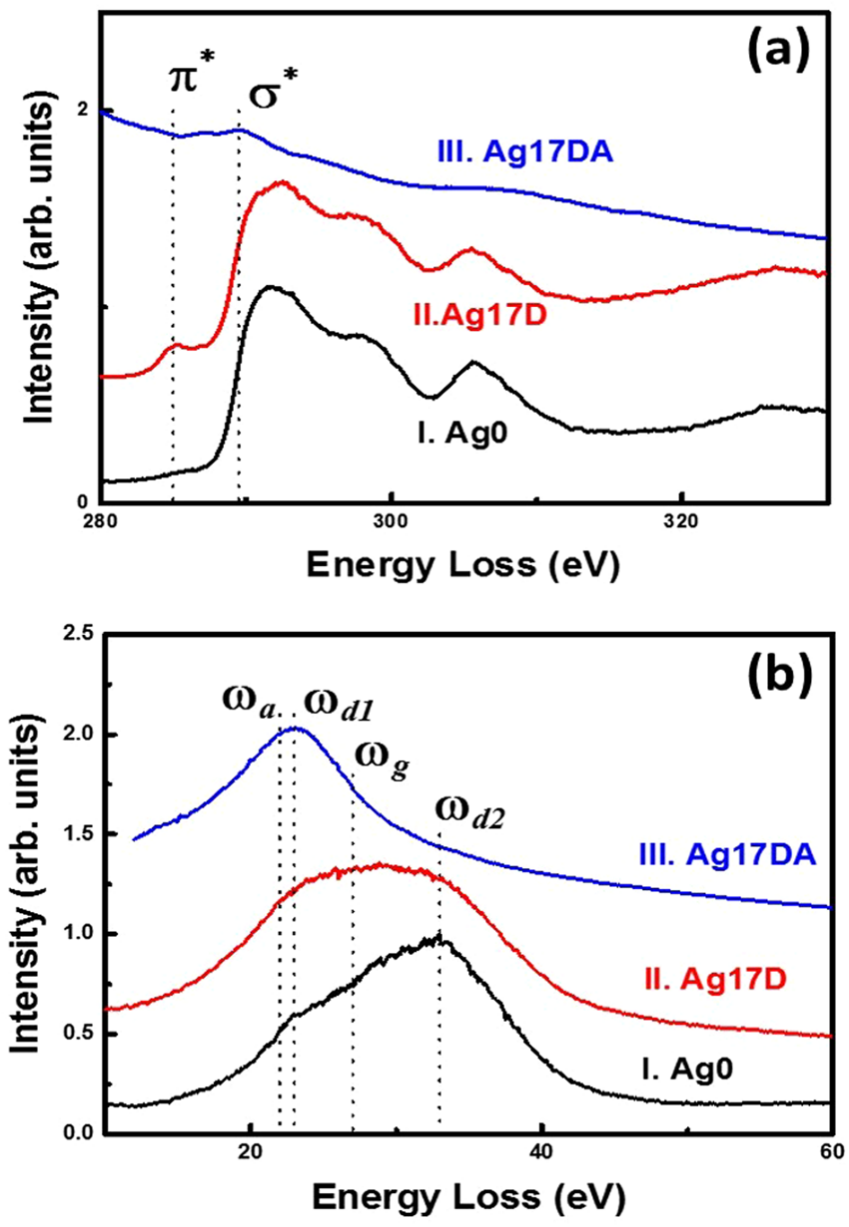
Selected area (**a**) core-loss EELS spectra and (**b**) plasmon-loss EELS spectra of the UNCD films, where (I) is Ag0, (II) is Ag17D, and (III) is Ag17DA.

The localized bonding structures of these materials were analyzed using the carbon K-edge EELS in TEM to clearly distinguish between the different carbon contents (e.g. diamond, graphite, and a-C)^44^. It should be mentioned that although the core-loss EELS is usually used for differentiating the presence of sp^3^-bonded carbon, the diamond, from the sp^2^-bonded ones (graphite and amorphous carbon), plasmon-loss EELS is the most effective measurement to differentiate the crystalline sp^2^-bonded carbon (graphite) from the amorphous carbon (a-C). The plasmon-loss EELS spectra for the graphitic phase shows a prominent peak at 27 eV (S_3_) and for the a-C phase at 22 eV (S_1_)^26,44,45^. The crystalline sp^3^-bonded carbon (i.e. diamond) show a plasmon-loss EELS peak at 33 eV (S_4_) corresponding to the bulk plasma resonance, with a shoulder peak at 23 eV corresponding to the surface plasma resonance^44,45^. Curve I in Fig. 4a shows the core-loss EELS spectra of the Ag0 films, indicating that these films contain a sharp peak at 289.5 eV (σ*-band) and a dip in the vicinity of 302 eV, which is the typical core-loss EELS signal for diamond (i.e. sp^3^-bonded carbon)^26,44^. The presence of a hump at 285 eV is very small, implying a small amount of sp^2^-bonded carbon contained in these Ag0 films. Moreover, the plasmon-loss EELS spectrum shown as curve I in Fig. 4b for Ag0 films contains a peak corresponding to the bulk plasmon resonance at S_4_ (33 eV) with a shoulder corresponding to surface plasmon loss at S_2_ (23 eV). The intensity ratio of I_S2_/I_S4_ is nearly 1/√2. This is, again, a typical characteristic of diamond materials. These EELS spectra confirm that the materials shown in Fig. 3a are predominantly diamond, even though most of the regions show low contrast as if they are not crystalline sp^3^-bonded carbon. Actually, they are diamond grains, which are oriented away from a zone-axis, thus weakly diffracting electrons. The TEM micrograph, diffraction patterns, and EELS spectra confirm that the Ag0 films are predominantly diamond materials.

In contrast, Fig. 3b and c shows the BF-TEM micrograph of the Ag17D and Ag17DA UNCD films, respectively, revealing that the structure of the pristine UNCD film was altered significantly because of the Ag-ion implantation and subsequent post-annealing processes. The large diamond aggregates that frequently occur in Ag0 films are no longer observable in the Ag17D and Ag17DA films, indicating that the energetic Ag-ions have disintegrated the soft diamond aggregates into ultra-small diamond grains. Aggregates of Ag particles about tens of nanometers in size are observed as spherical clusters in A17D films (indicated by arrows, Fig. 3b). The boundaries of the Ag clusters are rather sharp, implying that the Ag and carbon do not react with each other. Further, the SAED pattern in the inset of Fig. 3b illustrates that, besides the diffraction rings corresponding to the (111)D, (220)D, and (311)D lattice planes of diamond, there exist an extra diffraction ring corresponding to Ag (designated “Ag” in SAED). A large central diffuse ring was present in this SAED (inset, Fig. 3b), indicating that the Ag-ion bombardment has induced the formation of a significant proportion of sp^2^-bonded carbon for Ag17D films. The core-loss EELS spectrum (curve II, Fig. 4a) still shows a clear signature of sp^3^-bonded carbon (i.e., a sharp peak at 289.5 eV (σ*-band) and a dip in the vicinity of 302 eV). However, it contains a larger hump near 285 eV (π*-band), inferring the induction of a larger proportion of sp^2^-bonded carbon in Ag17D films due to the Ag-ion implantation process. The plasmon-loss EELS spectrum (curve II, Fig. 4b) reveals the presence of a large hump near 27 eV (S_3_-band) beside the S_2_ - and S_4_-bands corresponding to the diamond phase. This indicates that the sp^2^-bonded carbon induced by the Ag-ion bombardment is nanographitic in nature^26,44,45^.

Figure 3c, a typical BF-TEM micrograph for the Ag17DA films, shows an interesting phenomenon, that is, the diffraction rings corresponding to diamond materials are no longer observable in the corresponding SAED. Instead, randomly distributed diffraction spots are present along with a weak diffraction ring, corresponding to *i-carbon* (designated as *i*-C in inset I of Fig. 3c). Notably, the *i-carbon* is an allotropic phase of diamond with a bcc structure  (a_0_) = 0.432 nm)^44,46^. Several spherical particles on the order of hundreds of nanometers in size are present that do not seem to be crystalline materials (as no diffraction pattern was observed). A large central diffuse ring was observed in the SAED of the Ag17DA films (inset I, Fig. 3c), indicating that these films contain even more abundant sp^2^-bonded carbon compared with the Ag17D films. Occasionally, large Ag particles ~200 nm in size were observed (one of which is designated by an arrow in Fig. 3c). The enlarged TEM micrograph (inset II, Fig. 3c) and the associated diffraction pattern (inset III, Fig. 3c) indicate that this large particle is Ag. Apparently, these large Ag particles were formed via the coalescence of small Ag clusters present in the Ag17D films during the post-annealing process (cf. Figure 3b). The proportion of diamond particulates in these materials is relatively small compared with the *i-carbon* clusters and the matrix is mostly graphitic phase. In other words, most of the diamond grains in the Ag0 materials have been heavily damaged due to the high-dose Ag-ion implantation/post-annealing processes. The diamond grains were transformed toward a more stable phase (i.e. the graphitic phase). The *i-carbon* clusters are the metastable phase in the transformation routes from sp^3^-bonded carbon toward the more stable sp^2^-bonded carbon. The core-loss EELS spectrum of Ag17DA films (curve III, Fig. 4a) does not contain any feature of diamond (i.e., neither the σ*-band at 289.5 eV nor the secondary dip at 302 eV). Moreover, the plasmon-loss EELS spectrum (curve III, Fig. 4b) shows a single hump of relative large intensity at 22 eV, which corresponds to the a-C phase. This indicates that the materials contained in the Ag17DA films are mostly sp^2^-bonded carbon with amorphous phase. The bonding structure observed in the EELS (Fig. 4) is in accordance with the microstructural investigation shown in Fig. 3.

### PF-TUNA analysis of local electron field emission behavior of UNCD films

**Figure 5:**
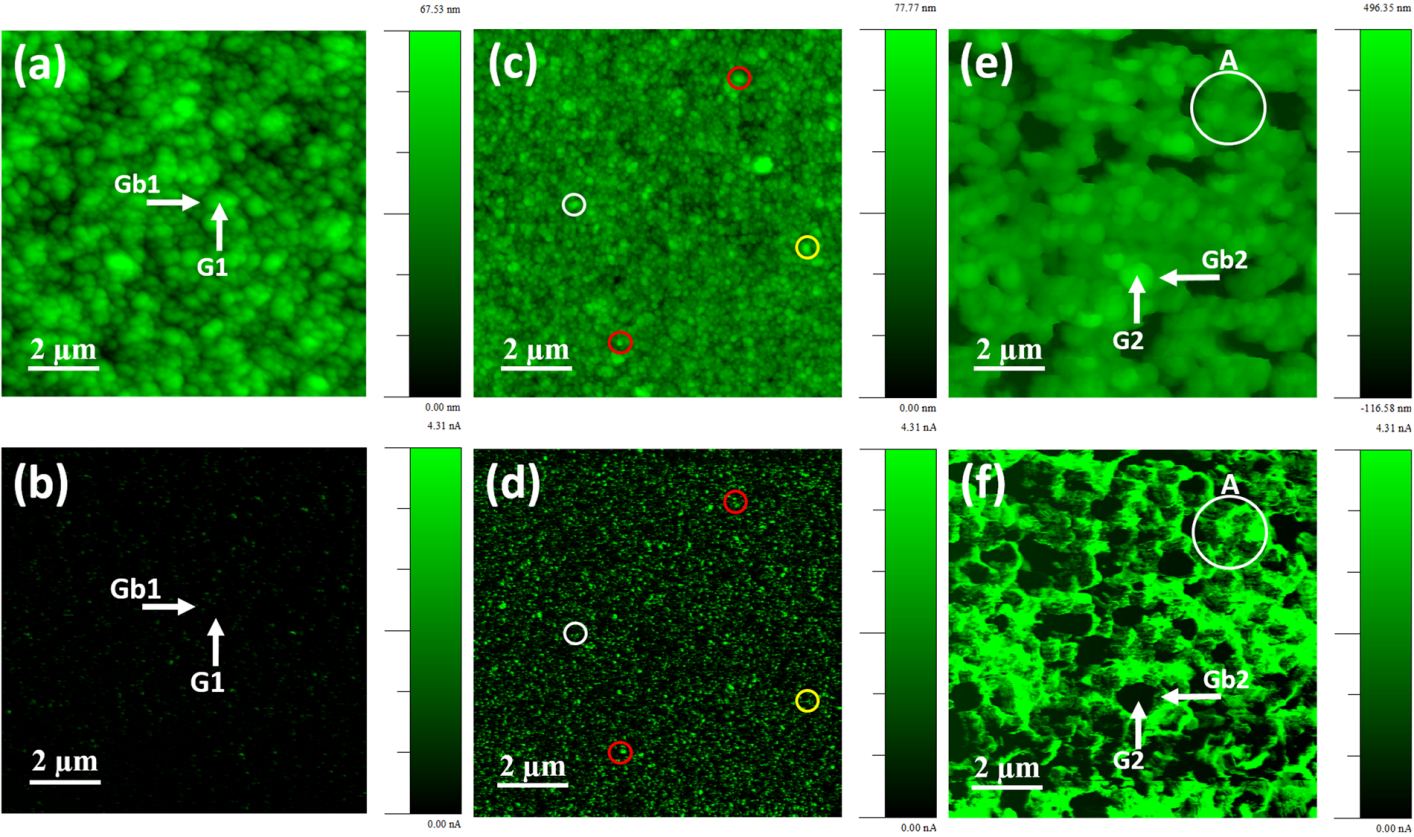
PF-TUNA images of the (**a**) Ag0, (**c**) Ag17D, and (**e**) Ag17DA films with their corresponding current mappings shown in (**b**,**d** and **f**), respectively, which were recorded at a tip bias of 2 V.

To understand how the Ag-ion implantation and subsequent post-annealing processes result in enhanced conductivity and EFE properties for these UNCD films, the local EFE properties of the Ag0, Ag17D, and Ag17DA films were investigated using AFM-based PF-TUNA technique. In the Ag0 films, the grains in the surface region of the samples coalesced and shows up as large diamond aggregate, which is marked as “G1” in Fig. 5a, whereas the grain boundary is marked as “Gb1”. Figure 5b is the PF-TUNA current mapping corresponding to Fig. 5a taken at a tip bias of 2 V. The shapes of the bright and dark regions in the PF-TUNA mapping in Fig. 5b are similar to those of the grain boundary “Gb1” and grain “G1”, respectively, in Fig. 5a. The bright contrast in the PF-TUNA image represents a higher current value with better electron emission properties. The PF-TUNA mapping in Fig. 5b reveals that there are very few electron emission sites for the Ag0 films, which are seen discontinuously throughout the sample surface.

A marked change in the surface morphology due to Ag-ion implantation was observed in the Ag17D films (Fig. 5c). The large diamond aggregates found frequently in the Ag0 films are no longer observed. Figure 5d shows the PF-TUNA current mappings (taken at the same tip bias of 2.0 V) corresponding to the respective surface morphology in Fig. 5c. Both the number density of the emission sites as well as the magnitude of the emission current in each site increased, as indicated in Fig. 5d.

Subsequent post-annealing of the Ag17D films (i.e. Ag17DA films) resulted in the formation of particulates, which are markedly larger than the UNCD clusters in the Ag17D films, as shown in Fig. 5e. Figure 5f illustrates that the emission sites with markedly larger emission current are preferentially seen along the boundaries surrounding the large particulates, which is marked “Gb2” in Fig. 5e. In contrast, the large particulates, marked “G2”, look dark in the PF- TUNA mapping, indicating that the large particulates are less emitting in nature compared with the boundaries between them. Notably, to compare the current of the emission site for these samples, the contrast in the PF-TUNA current mapping is adjusted to the same scale. Here it is to be mentioned that the higher lifetime stability of the Ag17DA films for cold cathode applications is illustrated by the evolution of the PF-TUNA emission current (RMS) against the time in Fig. S2 to illustrate the superiority of these materials compared with those of carbon nanotube, graphene and graphdiyne films^4–13^. The EFE properties of these carbon based nanomaterials is compared with the present Ag17DA films in Table 3.

**Table 3:** Comparison on the electron field emission properties of nano-carbon based field emitters.

Materials	Turn-on field (V/µm)	FEE current density	Lifetime stability (min)
Carbon nanotubes^4^	1.15	8.5 A/cm^2^ @ 4.6 V/µm	100
Carbon nanotubes on Inconel super alloy^5^	1.5	100 mA/cm^2^ @ 7 V/µm	—
Nanocrystalline diamond on Carbon nanotubes^6^	1.44	1.97 mA/cm^2^ @ 2.77 V/µm	33
Carbon nanotubes on nanopatterned substrate^7^	2.02	0.11 mA/cm^2^ @ 2.2 V/µm	80
Graphene sheets^8^	1.56	—	23
Few layer graphene sheets^9^	6	1 µA/cm^2^ @120 V/µm	120
Few layer graphene nanowalls^10^	1.2	—	100
Disordered manolayer graphene^11^	2.26	0.007 mA/cm^2^ @ 4.26 V/µm	—
Graphdiyne^12^	4.20	1.5 mA/cm^2^ @ 10 V/µm	78
Graphdiyne nanowalls^13^	6.6	2 A/cm^2^ @ 12 V/µm	—
Ag17DA^present study^	8.5	6.2 mA/cm^2^ @ 20.5 V/µm	660

**Figure 6:**
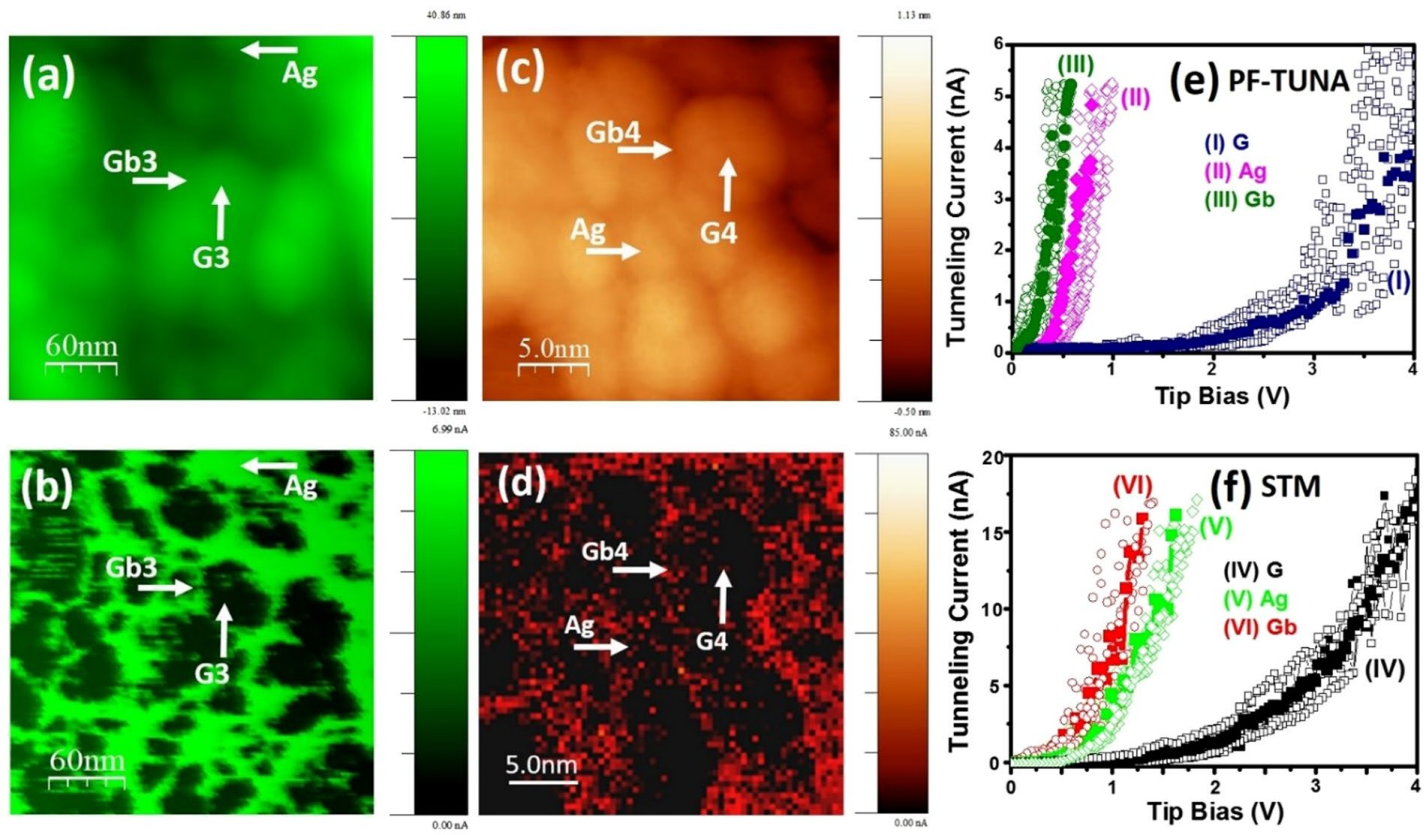
High-resolution local electron field emission characteristics for the Ag17DA films: (**a**) PF-TUNA surface morphology and (**b**) corresponding current mapping; (**c**) UHV high resolution STM image with corresponding (**d**) CITS mapping. (**e**) I–V characteristics curves recorded during (**e**) PF-TUNA and (**f**) STM scanning at the (I, IV) diamond grain (G), (II, V) Ag nanoparticles (Ag), and (III, VI) diamond grain boundary (Gb). The PF-TUNA and CITS images were acquired at a tip bias of 2.0 V.

The distribution of the emitting sites in the Ag17DA samples corresponding to region “A” of the samples (designated as circle in Fig. 5e) is illustrated by the high-resolution PF-TUNA surface morphology in Fig. 6(a) and PF-TUNA current mapping in Fig. 6b. Notably, the PF-TUNA current mapping (cf. Fig. 6b) shows more fine structure than the PF-TUNA surface morphology (cf. Fig. 6a). For example, the particulates 60 nm in size, designated “G3” in Fig. 6a, show a very uniform and smooth surface in the PF-TUNA surface morphology, but the PF-TUNA current mapping shows that some local areas emit more electrons than others. Such a phenomenon can be ascribed to the fact that the said particulates actually consist of smaller clusters and the emitting sites are locating at the boundaries between these ultra-small clusters. These observations imply that the resolution of the PF-TUNA current mapping is better than the PF-TUNA surface morphology. It should be noted that, in the Ag17DA samples, some clusters also emit electrons strongly like the grain boundaries. A typical cluster is marked “Ag” in Fig. 6a with the corresponding PF-TUNA current mapping marked in Fig. 6b. This region is marked as “Ag” because the TEM investigations suggested the existence of Ag nanoparticles (cf. Fig. 3c). The PF-TUNA current mapping of the Ag17DA surfaces in Fig. 6b indicates that the grain boundaries form interconnected conducting sites, such that the electrons can travel along these conducting grain boundary sites easily.

Moreover, to illustrate the advantage of the AFM-based PF-TUNA current mapping over STM based CITS mapping in the characterization of local EFE behavior, high resolution ultra-high vacuum STM (HR UHV-STM) surface morphology along with the corresponding CITS mapping were recorded for the Ag17DA surfaces and are shown in Fig. 6c and d respectively. CITS mapping corresponding to a typical diamond grain marked “G4” with the grain boundary “Gb4” (Fig. 6c) is illustrated in Fig. 6d. This micrograph shows better electron emission along the grain boundaries (Gb4), as it is of brighter contrast. Here, it is seen that the UHV STM image has better resolution than the ambient PF-TUNA measurement that can clearly show the ultra-small diamond grain and grain boundaries for the Ag17DA films. However, the STM based CITS mapping does not have good enough contrast to clearly illustrate the distribution of the local electron emission sites. The prime factor for such a deficiency is that, in STM mode, the feedback circuit does not allow for precise positioning of the STM tip and does not guarantee the same tip-to-sample distance during the acquisition of the emission current. Nevertheless, both PF-TUNA and CITS measurements revealed that the grain boundaries are more prominent electron emission sites compared with diamond grains (or nano-sized clusters) for the Ag17DA films.

Furthermore, local current–voltage (I–V) characteristic curves in the AFM-based PF-TUNA and STM-based CITS for the Ag17DA films were acquired to crosscheck the local electron emission mappings recorded by the respective techniques. The local I–V curves in the PF-TUNA measurements are illustrated in Fig. 6e as diamond grains (labelled “G”, curve I), Ag nanoparticles (labelled “Ag”, curve II) and grain boundaries (labelled “Gb”, curve III). Similarly, the local I–V curves recorded while scanning the UHV STM image for the Ag17DA samples are shown in Fig. 6f at various sample positions as on the grains (labelled “G”, curve IV), Ag nanoparticles (labelled “Ag”, curve V), and grain boundaries (labelled “Gb”, curve VI). It should be mentioned that corresponding to each emission site, eight reproducible I–V curves were recorded during scanning, which are shown as open symbols, and the averages of these curves are plotted as solid symbols. The local I–V curves for the UHV STM show more fluctuating data points, which is, again, attributed to the uncertainty in controlling the height of the STM tip. Interestingly, both the PF-TUNA and HR STM measurements reveal a significant difference in the I–V characteristic curves at the grain and grain boundaries. The grain boundaries (curve III in Fig. 6e and curve VI in Fig. 6f) start to emit at a lower sample bias compared with the grains (curve I in Fig. 6e and curve IV in Fig. 6f). In both measurements, a few Ag nanoparticles marked as “Ag” in Fig. 6a and c, are also seen to emit high current at a lower sample bias, like the grain boundaries, which are illustrated as curve II in Fig. 6e and curve V in Fig. 6f. The local I–V curve measurements from both the PF-TUNA and UHV STM techniques agree well with the phenomena that the emission current is prominent along the grain boundaries and Ag nanoparticles compared with the grains. It should be noted that, although the scanning probe microscopy and electron field emission measuring schemes are not directly equivalent, the tunnelling measurements by PF-TUNA can help to better understand and interpret the field emission behaviour taken from a very local scale for diamond and related materials.

### Catalytic phase transformation mechanism for the formation of nanographitie phase

The PF-TUNA, CITS, and local I–V measurements clearly illustrate that electron emission from Ag-ion implanted/post-annealed UNCD surfaces originate preferentially from the grain boundaries and not from the diamond grains or other topographical features in the materials. These experimental results of grain boundary EFE behavior also support the findings of other groups^47–51^. As long as the grain boundaries remain comparatively more conductive than the bulk grains, electrons can travel from the electoral contact at the reverse side of the Si substrate into the base of the film through the grain boundaries up to the top surface of the materials and are then easily field emitted to the vacuum.

**Figure 7:**
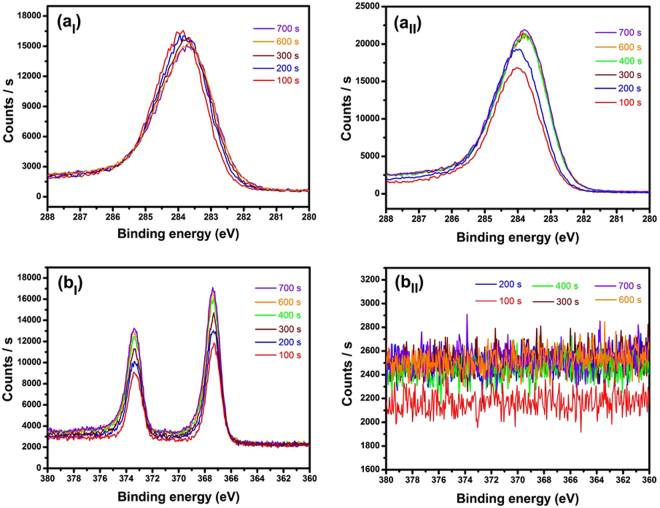
X-ray photoelectron spectroscopy depth profile characterization of (a_I_,a_II_) C 1 s and (b_I_,b_II_) Ag 3d peaks obtained after each etch cycle of 100 s to a depth of ~47 nm (700 s ion sputtering) inside the (a_I_,b_I_) Ag17D and (a_II_,b_II_) Ag17DA films recorded during etching using an Ar ion gun (at 2 kV and 1 mA). (Note: few spectra are not shown here to provide more clarity, as they almost merge with each other).

The mechanism by which the Ag-ion implantation/post-annealing process enhanced the conductivity and EFE properties of the UNCD films can be accounted for by the graphitization of the a-C phases along the grain boundaries. Such a proposed mechanism was supported by previous reports that the incorporation of some metallic species into carbon films can act as catalysts for the conversion of sp^3^-bonded carbon to sp^2^-bonded carbon, and the post-annealing process is the key step for inducing the graphitization of the a-C phase^52–62^. To elucidate the genuine mechanism, by which the implanted Ag-ions can induce the formation of the nanographite phases, the XPS depth profiling results for the C1s and Ag 3d spectra of Ag17D and Ag17DA films were investigated. The XPS spectra shown in Fig. 7 clearly show the presence of C1s (Fig. 7(a_I_)) and Ag 3d (7(b_I_)) core level spectra on the subsurface and deep inside the surface after each etching cycle of the Ag17D films. The spectra remain similar in structure as we move from the surface to a depth of ~47 nm below the surface (i.e. for films sputtered for 700 s). This clearly indicates that the UNCD films are implanted with Ag ions. However, for Ag17DA films (i.e. post-annealed Ag17D films), while the C1s peak is still clearly observable, no such Ag 3d spectra were recorded, as shown in Fig. 7(a_II_) and (b_II_), respectively.

This indicates that the Ag ions do not directly react with the carbon species in the implanted/post-annealed UNCD films, but diffused and redistributed uniformly all over the UNCD films, forming Ag clusters at much smaller concentrations (almost undetectable). A large proportion of the nano-graphitic phase was induced, probably via the dissociation – absorption ‒ reprecipitation mechanism, which is similar to the one proposed by Berman *et al*. for the catalytically formation of the nanographitic phase on Ni-coated UNCD films^63^. Notably, in Berman’s model, the Ni clusters catalytically dissociated the UNCD at temperatures around 900 °C, absorbed the carbon species into the Ni clusters, and reprecipitated them out during cooling, resulting in the formation of graphene on the surface of these Ni clusters.

**Figure 8:**
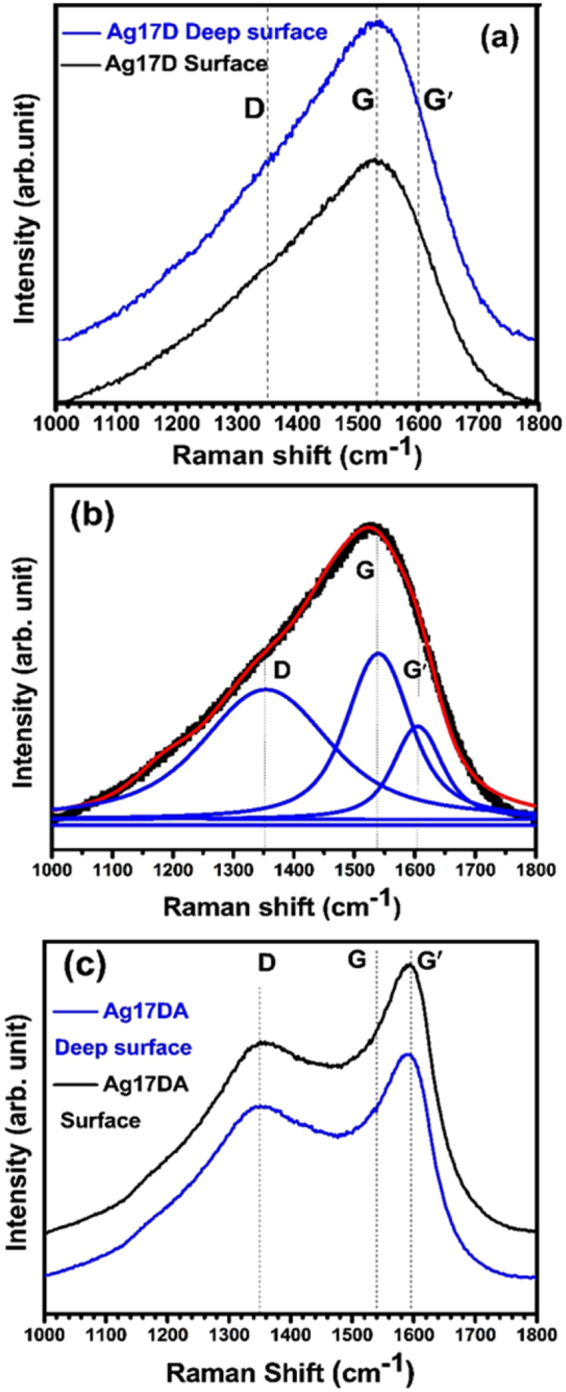
Raman spectra (**a**) on both surface and deep inside the Ag17D films (**b**) Deconvolution of the spectra obtained from the surface of the Ag17D films and (**c**) Spectra both at the surface and deep inside the Ag17DA films.

Moreover, to double-check the formation of nanographitic phase on the surface of both Ag ion implanted (Ag17D) and post-annealed (Ag17DA) UNCD films as observed by XPS and TEM measurements, the Raman spectra were recorded. The Raman spectra were measured on the Ag17D and Ag17DA film’s surfaces and ~47 nm below the surface (for 700 s sputtered films inside XPS chamber) as described in Fig. 8. Interestingly, we observed no change in the Raman spectra on the surface and deep inside the surface of the Ag17D and Ag17DA films as shown in Fig. 8a and c respectively. The Raman spectra recorded on the surface and ~47 nm deep inside the surface of Ag17D, showed the presence of a large hump around 1540 cm^−1^ as shown in Fig. 8b. Deconvolution of this hump (Fig. 8b) reveals that, the disordered diamond (D ~ 1350 cm^−1^), graphitic (G ~ 1540 cm^−1^), and nanographitic (G′ ~ 1600 cm^−1^) phases occur both at the surface and deep inside the surface, with the dominant graphitic phase. Moreover, the Raman spectra recorded at the surface and below the surface of the Ag17DA films (Fig. 8c) clearly show the presence of disordered diamond (D ~ 1350 cm^−1^), graphitic (G ~ 1540 cm^−1^), and a prominent nanographitic (G′ ~ 1600 cm^−1^) peak. It shows the defective graphitic phase (G peak), observed in Fig. 8b converted into a more stable prominent nanographitic phase after the post-annealing process.

The formation of these nanographitic phase surrounding the nano-sized diamond grains of the UNCD resulted in an interconnected conduction network that enriched the electrical conductivity and the EFE properties of the UNCD films. Thus, the Raman spectra clearly support the TEM and XPS results that the nanographitic phase was enhanced after the post-annealing of the Ag ion implanted UNCD films.

Notably, although the implanted Ag ions did not directly react with the carbon species in the UNCD films, significant damage of the diamond lattice was induced by the bombardment of heavy and energetic Ag ions, which resulted in the formation of *i-carbon* clusters or even directly transform the diamond grains into non-diamond clusters. It should be mentioned that the *i-carbon* with a bcc structure is actually the defected diamond lattice, in which some of the carbon atoms in the diamond lattices are missing. Moreover, the non-diamond clusters were formed when too many of the carbon atoms in the diamond lattice were displaced. However, the conductivity of the material can be maintained at a high level as long as the grain boundaries contain abundant nanographitic phase. In such a situation, whether the ultra-small diamond grains remain as perfect sp^3^-bonded carbon or have been converted into *i-carbon* clusters (or other non-diamond carbon clusters) is immaterial. These observations are in accord with the conducting mechanism proposed for the UNCD films, viz. the grain boundaries are the electron conducting path and electron field emitting sites for all Ag0, Ag17D, and Ag17DA materials.

## Conclusions

In summary, first, a feasible way to fabricate high-conduction UNCD films with enhanced field emission properties by a simple Ag-ion implantation/annealing process is demonstrated. Second, the mechanism for the enhanced field emission properties of the Ag-ion implanted diamond films were revealed locally by PeakForce-controlled AFM investigations by mapping the nanoscale electron emission sites, which demonstrated that the grain boundaries are the prominent field emission sites. Detailed nanoscale investigations of the origin behind the enhanced electron emission sites were performed by UHV STM, which support the AFM-based PF-TUNA results. Both SPM-based techniques, CITS and PF-TUNA mapping, directly revealed that these interconnected conducting grain boundary channels throughout the Ag-ion implanted/annealed UNCD surfaces give rise to prominent electron emission sites, resulting in higher electrical conductivity and enhanced EFE properties. Finally, the formation of nanographitic phases along the diamond grain boundaries, confirmed from TEM examination, seem to improve the conductive nature by easy transport of electrons that are possibly the main factor for the enhanced conductivity and field emission properties of the Ag-ion implanted/annealed UNCD films.

A final conclusion from this study is that designing diamond films into sharp cones, needles, or nanowire structures to enhance the conductivity/field emission properties is not required. Conductive diamond surfaces with a large number of graphitic grain boundary channels, metal ion-implanted diamond surfaces, or chemical functionalization can efficiently transform the diamond to a sp^2^-graphitic phase and can provide excellent field emission sources, even from a flat surface. Moreover, the fabrication of these films with higher electrical conductivity/superior field emission properties will certainly open up new prospects for flat-panel displays and high-brightness electron sources.

## Methods

UNCD films were grown on *n*-type silicon substrates in a microwave plasma-enhanced chemical vapor deposition (2.45 GHz IPLAS-CYRANNUS-I, Troisdorf, Germany) system. Prior to UNCD film growth, the Si substrates were thoroughly rinsed sequentially using a water-diluted hydrogen peroxide/ammonium hydroxide solution and then a hydrogen peroxide/hydrochloric acid solution. The silicon substrates were then ultrasonicated in a methanol solution containing a mixture of nanodiamond powder (about 4 nm in size) and titanium powder (<32.5 nm) (SIGMA-ALDRICH; 365 mesh) for 45 min to facilitate the nucleation sites for growing the UNCD films. The UNCD films were grown on these pre-treated Si substrates in an Ar (99%)/CH_4_(1%) plasma (100 sccm), at 1200 W and 200 mbar for 3 h without intentional heating of the Si substrate. The substrates were heated to around 450 °C by plasma ion bombardment that was measured by a thermocouple embedded in the stainless steel substrate holder. Silver ions were then implanted into the UNCD films by an ion implanter machine (1.7 MV Tandetrol Accelerator) at room temperature with an implantation energy of 400 keV and ion dosages varying from 1 × 10^15^ to 1.74 × 10^17^ ions cm^−2^. The pristine UNCD films are designated as Ag0 and the films implanted with the Ag ion doses of 1 × 10^15^, 1 × 10^16^, 5 × 10^16^, 1 × 10^17^, and 1.74 × 10^17^ ions cm^−2^ are designated as Ag15, Ag16, Ag5 × 16, Ag17, and Ag17D, respectively. TRIM software^64^, was used to evaluate the trajectory of the ions and to estimate the critical dose for transforming the sp^3^ bonds to sp^2^ bonds^65^. The ion range and straggling of the implanted ions, calculated by TRIM, are 102.4 nm and 19.2 nm, respectively, as shown in Fig. S3b and c in the supplementary information. The Ag17D films were subsequently annealed at 600 °C in a N_2_ gas atmosphere for 30 min and are designated as Ag17DA. The surface morphology of these films was examined using field emission scanning electron microscopy (FESEM, Joel 6500). The chemical bonding structure of the films was investigated using X-ray photoelectron spectroscopy (XPS; PHI 1600). XPS depth profiling was carried out at sputtering times of 100‒700 s (at 100 s intervals and a sputtering rate of ~4 nm in 60 s) to a depth of ~47 nm below the sample surface (i.e. sample sputtered for 700 s) by the Ar ion etching system attached to the UHV XPS system. We first standardize the Ar ion etching system to know the sputtering rate for both the Ag17 and Ag17DA films and then we calculated the depth at which the XPS spectra were recorded after a particular sputtering time period. The detailed microstructures and the bonding structures of these films were examined using transmission electron microscopy (TEM, Joel 2100 F) and electron energy loss spectroscopy (EELS, Gatan Enfina) in TEM, respectively.

To examine the conducting behavior of these films, Hall measurements were carried out in the van der Pauw configuration (ECOPIA HMS-3000). The EFE measurements were carried out in a tunable parallel plate setup, in which the gap between the UNCD films (cathode) and molybdenum tip (anode, 2 mm diameter) is controlled by a micrometer. The field emission currents were noted for each applied voltage using an electrometer (Keithley 2410) at a pressure of about 1 × 10^−6^ mbar. PF-TUNA measurements were performed using a multi-mode VIII AFM with a Nanoscope V controller and PF-TUNA module [Bruker, CA, USA] at ambient conditions. The bases of the Si substrates were attached to a steel disc using conductive silver paint (G3790 Agar Scientific) to make the electrical contact between the UNCD films and the AFM tip. It should be noted that in PF-TUNA, the surface is scanned in tapping mode to get the morphology of the sample surface along with the average current value throughout the scanned area which is used to get the PF-TUNA current mapping. The tip taps the surface within a distance of few nm, which can be controlled using a sensitive feedback system known as PeakForce control that maintains an air gap between the AFM tip and the sample. Specifying a force level < 1 nN as the set-point ensures that the tip–sample interaction remains in the weakly attractive region of the Lennard–Jones potential^66^, thus the separation can be maintained at a few nanometers. The field emission currents, as opposed to the surface current are acquired. In PF-TUNA, the topographic and tunneling current information are collected using PeakForce feedback (i.e. a non-harmonic AFM feedback that oscillates the sample and uses the resulting cantilever–force curves to maintain a set-point force level). The force level between the conductive tip and the sample is maintained in such a way that tunneling current measurements are possible with a minimum 1 nm separation between the tip and the sample. Therefore, this technique has an additional benefit over contact-mode AFM in preventing the tip from being damaged by the sample. This is a crucial factor when investigating a hard sample (*e*.*g*. diamond) or when scanning large features or high-RMS roughness surfaces. The control of parameters like non-harmonic PeakForce control and force level—and thus the separation between the tip and the sample—can be maintained more precisely, allowing PF-TUNA measurements and surface topography to be collected simultaneously in a non-contact regime. The PF-TUNA technique is found to be a more representative picture of the field emission scenario with the additional advantage of performing the experiment at a very local scale on the sample surface to detect the exact electron emission sites.

Topographic and tunneling current information were collected using PeakForce feedback. A Pt‒Ir coating on the tip of the cantilever, with a spring constant of 9.8 N/m, allows the measurement of current when a bias is applied between the tip and the sample. Images were recorded at a resolution of 512 × 512 pixels with a tip bias of a few volts at a lower scan rate (usually 0.1 or 0.2 Hz) to allow the maximum time for the TUNA current measurement. A variety of tests were performed in this scenario to confirm that the measured TUNA current was a true reflection of the EFE properties of the surface and not from any artifacts present on the surface. The recorded EFE currents were in the range of a few pA to nA per emission site, with tip–sample biases varying from mV to V. Moreover, to cross check the PF-TUNA results, a commercial ultrahigh vacuum scanning tunneling microscopy (UHV STM, 150 Aarhus, SPECS GmbH) system was used at room temperature with a base pressure of at least 5 × 10^−10^ Torr. Current image tunneling spectroscopic (CITS) spectra with voltages ramping from −3 to +3 V were measured simultaneously during the STM imaging. Tungsten tips, which were prepared using the electrochemical etching process, were used for the topographic imaging and CITS mapping.

## Electronic supplementary material

Supplementary Information 
