# Supplementary material for: Nanoscale investigation of enhanced electron field emission for silver ion implanted/post-annealed ultrananocrystalline diamond films
Source: Sci Rep. 2017 Nov 24;7:16325. doi: 10.1038/s41598-017-16395-1 (PMC5701233; doi:10.1038/s41598-017-16395-1)
Supplement: Supplementary file 1 — Supplementary Information [file 41598_2017_16395_MOESM1_ESM.doc]

**Supplemental Information for**

**Nanoscale investigation of enhanced electron field emission for silver ion implanted/post-annealed ultrananocrystalline diamond films**

Kalpataru Panda^1, †^, Jin Hyeok Jeong^2^, Jeong Young Park^1,2,†^, K. J Sankaran^3,4^, B. Sundaravel^5^, I-Nan Lin^6^

^1^Center for Nanomaterials and Chemical Reactions, Institute for Basic Science (IBS), Daejeon, 34141, Korea

^2^Graduate School of EEWS, Korea Advanced Institute of Science and Technology (KAIST), Daejeon, 34141, Korea

^3^Institute for Materials Research (IMO), Hasselt University, 3590, Diepenbeek, Belgium

^4^IMOMEC, IMEC vzw, Diepenbeek, Belgium

^5^Materials Physics Division, Indira Gandhi Centre for Atomic Research, Kalpakkam 603 102,

India

^6^Department of Physics, Tamkang University, Tamsui 251, Taiwan, ROC


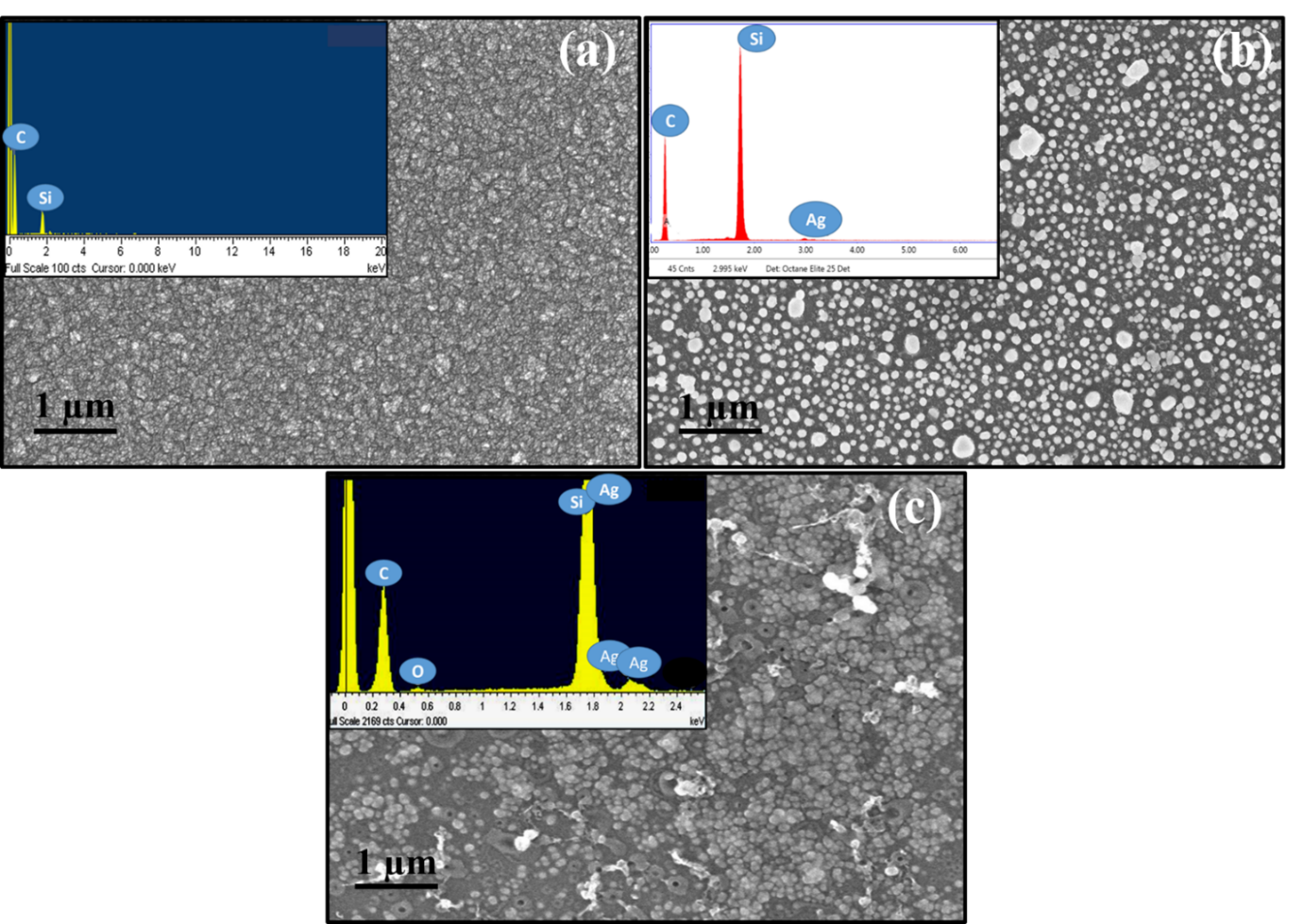


**Figure S1.** FESEM images of the (a) Ag0, (b) Ag17D, and (c) Ag17DA films. The inset shows the respective EDX mapping that indicates the presence of Ag on the (b) Ag17D and (c) Ag17DA films after Ag-ion doping and the subsequent post-annealing process.


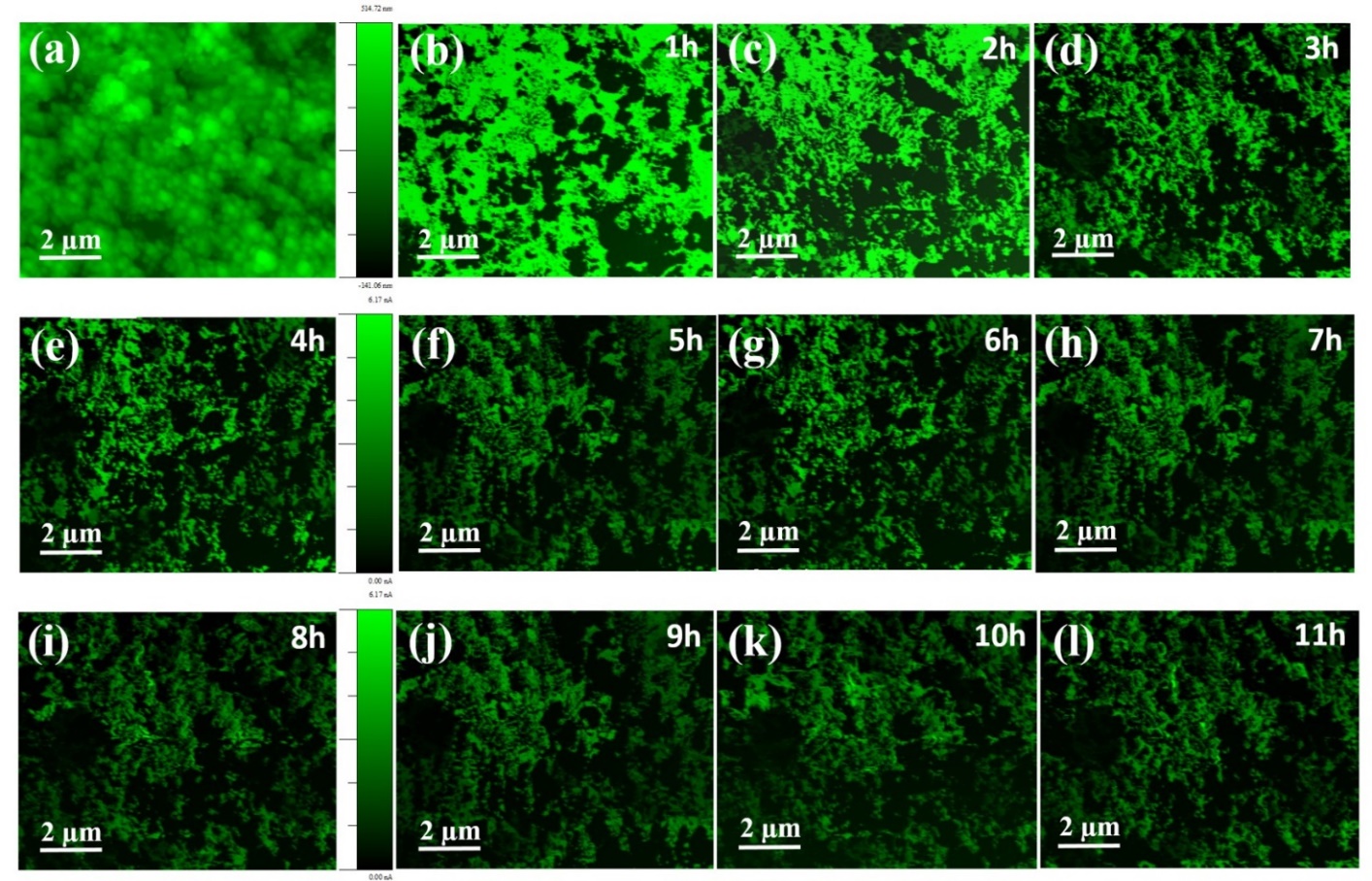


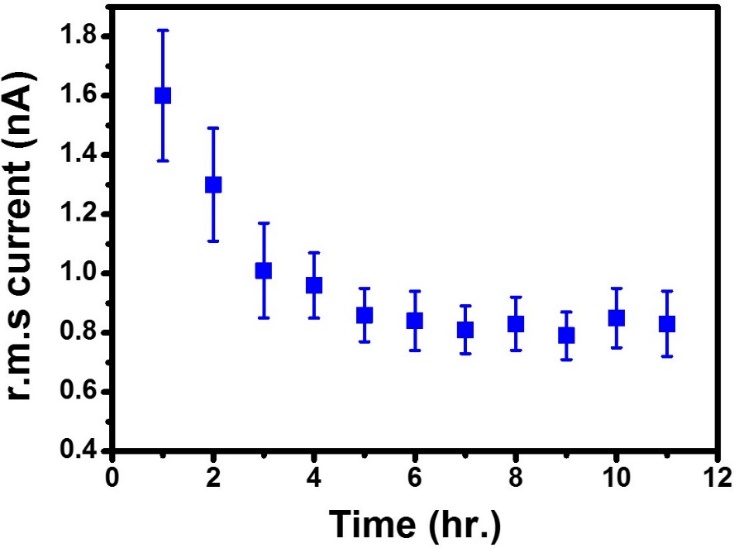


(m)

**Figure S2.** Measured field emission current mapping (10×10 µm) on the Ag17DA surface by PF-TUNA for a period of 11 hrs. The scanning conditions (i.e*.* scan rate, number of lines/scan) are adjusted such that each PF-TUNA current mapping takes 1 h. (a) Surface topography, (b) 1 h after loading the sample in the PF-TUNA set up, and similarly (c) 2 h, (d) 3 h, (e) 4 h, (f) 5 h, (g) 6 h, (h) 7 h, (i) 8 h, (j) 9 h, (k) 10 h, and (l) 11 h. The images have been color-scaled from dark green (low emission) to light green (high emission). The lower brightness of (d–l) compared with (a and b) indicates that the emission intensity decreased after the first 2 h time period. After the first 2 h, the field emission current is found to be stable for the subsequent 8 hrs. (m) Evolution of the PF-TUNA emission current (RMS) measured against time extracted from PF-TUNA current mapping, Fig. (a‒l) on the sample surface.

**(a)**

**(b)**

**(c)**


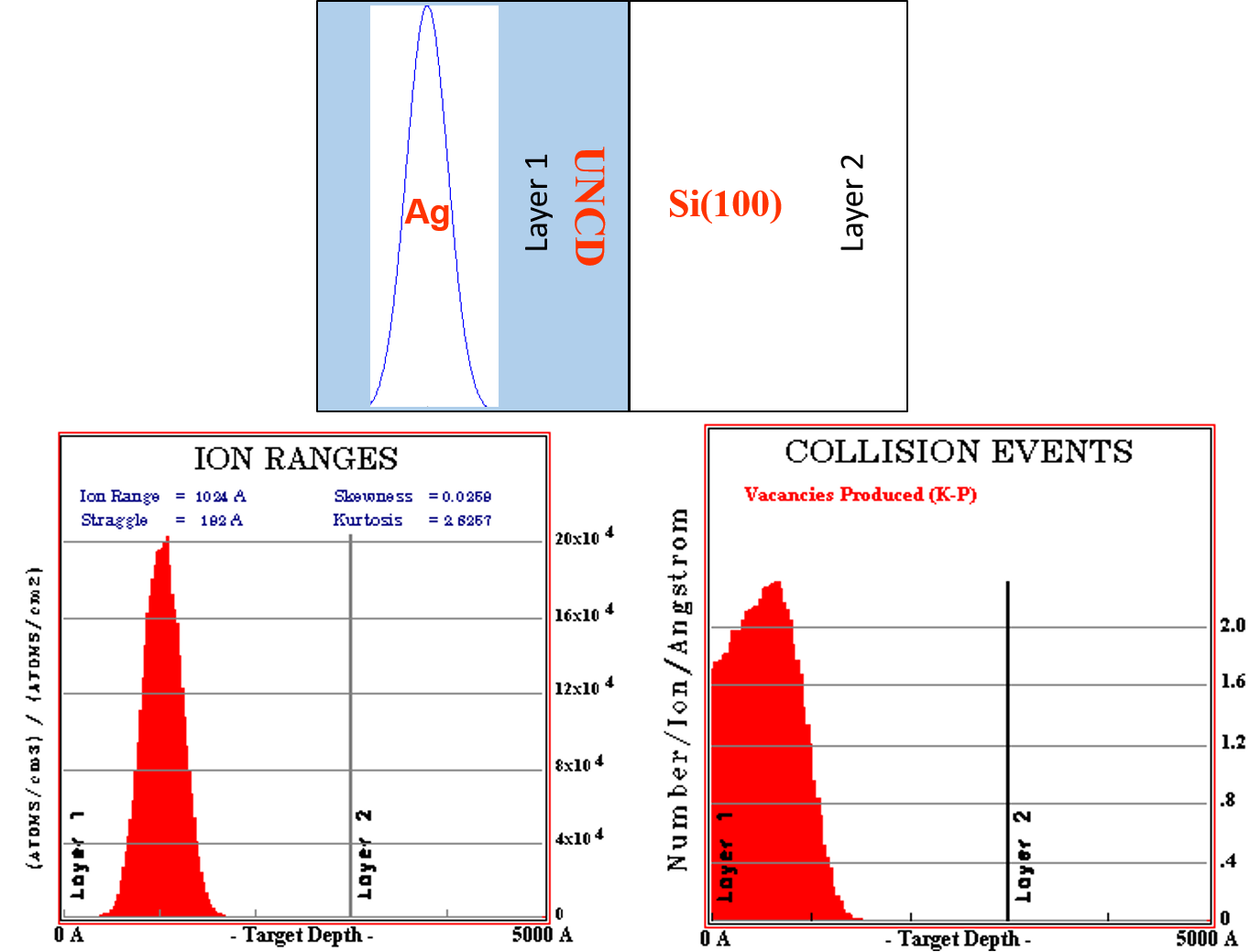


**Figure S3.** TRIM software calculation to evaluate the trajectory of ions (e.g. ion range and straggling) in the UNCD films. (a) Schematic figure demonstrating a picture of the implanted Ag ions in the UNCD films deposited on a Si substrate. (b) Calculated results from the TRIM software with an ion range of 102.4 nm and straggling of 19.2 nm. (c) The ion energy was chosen such that the Ag ion implantation only affected layer 1 of the UNCD films because vacancies are only seen in that region.
